# Supplementary material for: Network vulnerability of cattle movement in Minas Gerais, Brazil, from 2013 to 2022
Source: PLoS One. 2025 Dec 1;20(12):e0317275. doi: 10.1371/journal.pone.0317275 (PMC12668548; doi:10.1371/journal.pone.0317275)
Supplement: S2 Function — https://github.com/anninhactrbc/ntw_vulnerability_super_spreaders_susceptible.git. (PDF) [file pone.0317275.s002.pdf]

S2: Super spreaders and super susceptible function.

https://github.com/anninhactrbc/ntw_vulnerability_super_spreaders_susceptible.git
