# Supplementary material for: Network vulnerability of cattle movement in Minas Gerais, Brazil, from 2013 to 2022
Source: PLoS One. 2025 Dec 1;20(12):e0317275. doi: 10.1371/journal.pone.0317275 (PMC12668548; doi:10.1371/journal.pone.0317275)

S3: The proportion of nodes with the highest value of betweenness that most significantly contributes to reducing the region's vulnerability evaluated over the following years (from one to nine) to quantify the sustainability of targeted surveillance based on the ranking of previous years.

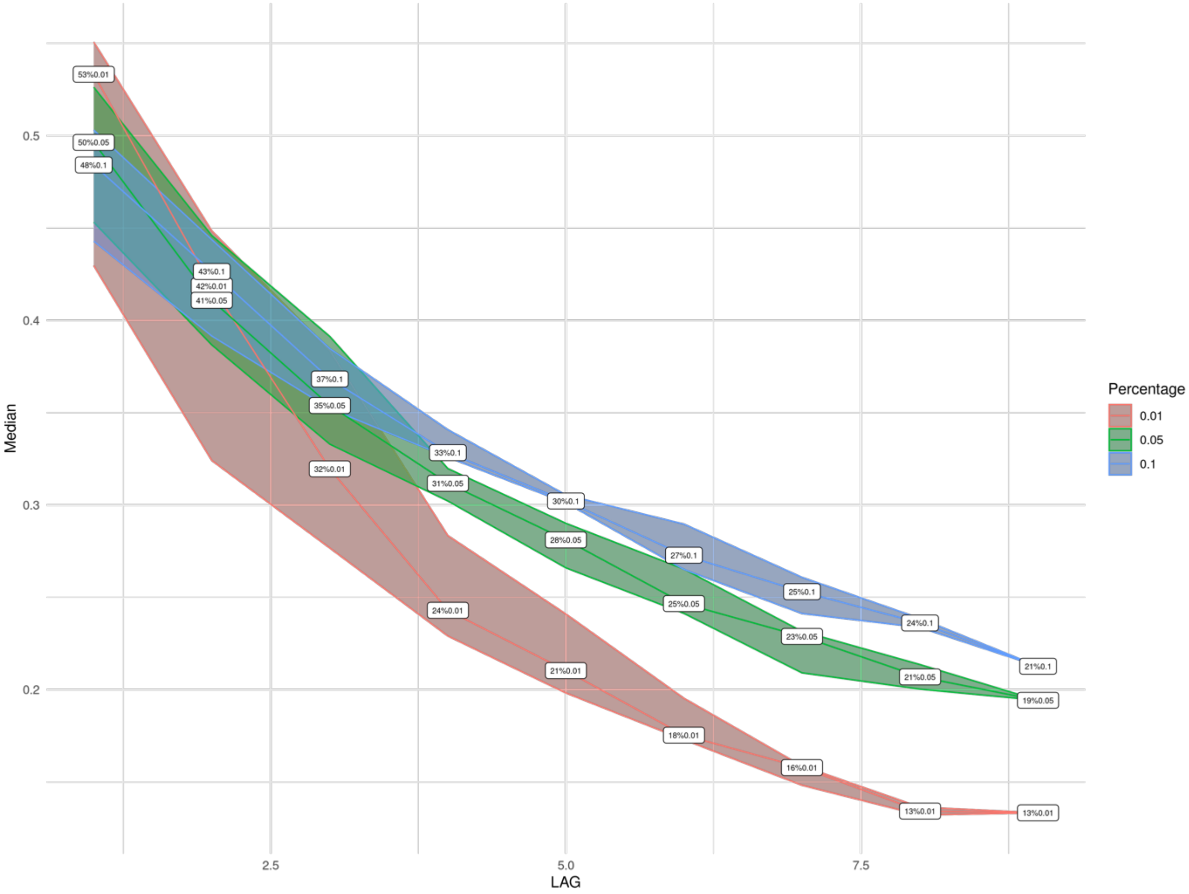

Supplement: S3 Fig — (PDF) [file pone.0317275.s003.pdf]
