# Supplementary material for: Network vulnerability of cattle movement in Minas Gerais, Brazil, from 2013 to 2022
Source: PLoS One. 2025 Dec 1;20(12):e0317275. doi: 10.1371/journal.pone.0317275 (PMC12668548; doi:10.1371/journal.pone.0317275)
Supplement: S4 Fig — The colored lines are the regions, and the light green represents the less possible vulnerability. The regions are 01: Northwest Minas, 02: North Minas, 03: Jequitinhonha, 04: Vale do Mucuri, 05: Triângulo Mineiro/Alto Paranaíba, 06: Central Minas, 07: metropolitan area of Belo Horizonte, 08: Vale do Rio Doce, 09: West Minas, 10: South/Southeast Minas, 11: Campo das Vertentes and 12: Zona da Mata. (PDF) [file pone.0317275.s004.pdf]

**S4 Fig: Network Vulnerability of cattle movement per month and region in Minas Gerais state, Brazil in 2022.** The colored lines are the regions, and the light green represents the less possible vulnerability. The regions are 01: Northwest Minas, 02: North Minas, 03: Jequitinhonha, 04: Vale do Mucuri, 05: Triângulo Mineiro/Alto Paranaíba, 06: Central Minas, 07: metropolitan area of Belo Horizonte, 08: Vale do Rio Doce, 09: West Minas, 10: South/Southeast Minas, 11: Campo das Vertentes and 12: Zona da Mata.

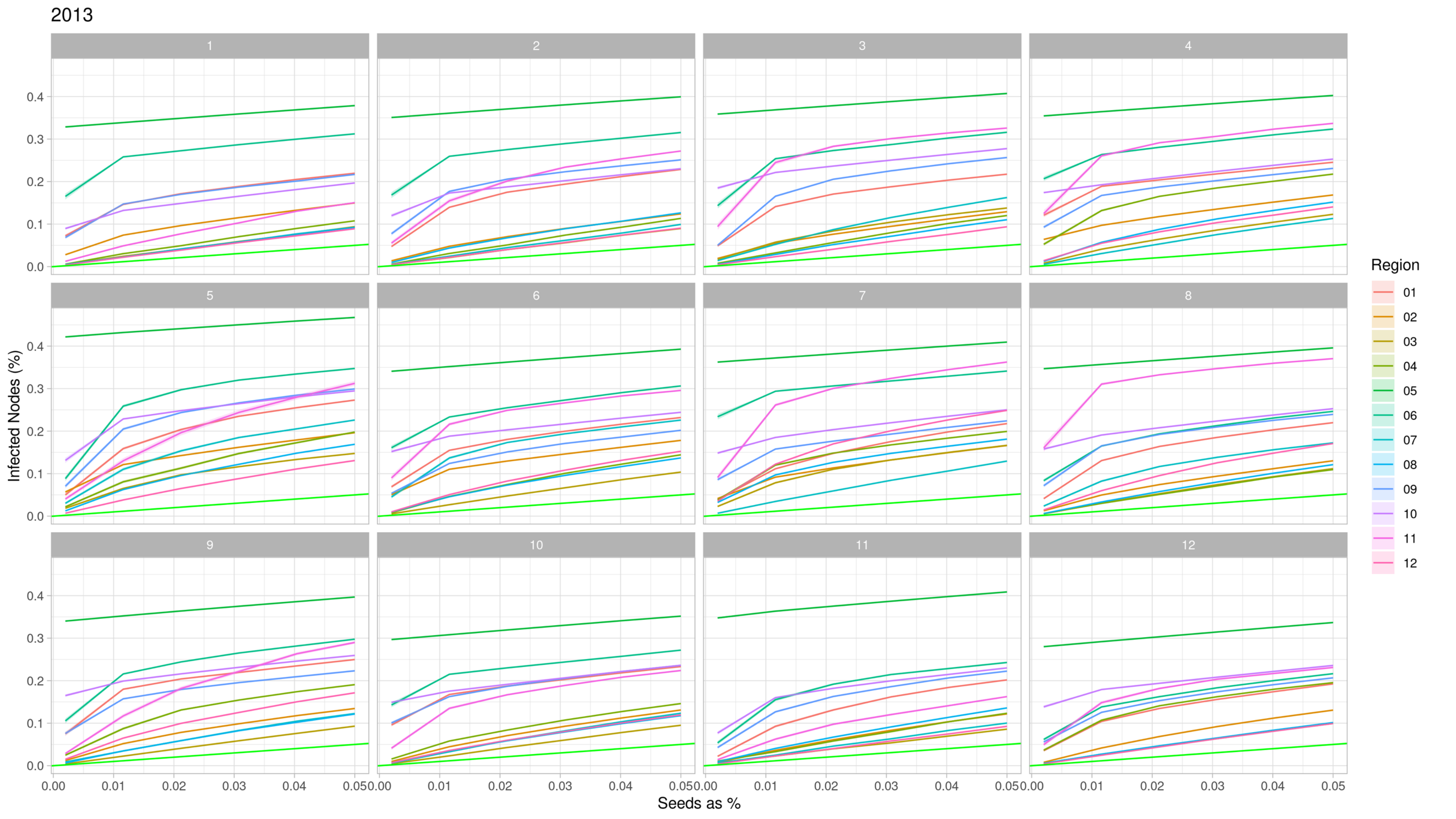

2013
1
2
3
4
5
6
7
8
9
10
11
12
0.0
0.1
0.2
0.3
0.4
0.00
0.01
0.02
0.03
0.04
0.05
Infected Nodes (%)
Seeds as %
Region
01
02
03
04
05
06
07
08
09
10
11
12

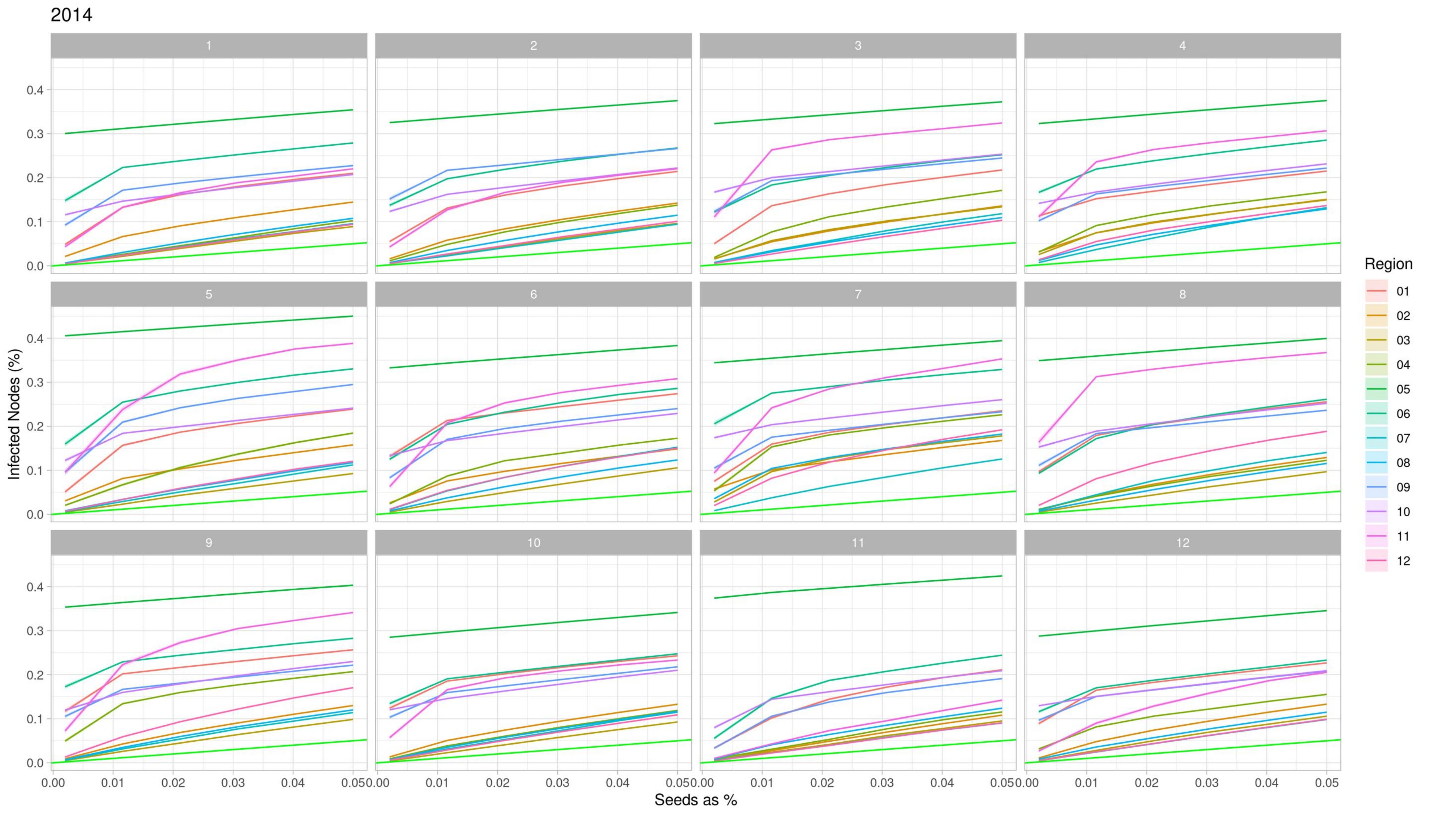
2014
1
2
3
4
5
6
7
8
9
10
11
12
Infected Nodes (%)
Seeds as %
0.0
0.1
0.2
0.3
0.4
0.00
0.01
0.02
0.03
0.04
0.05
Region
01
02
03
04
05
06
07
08
09
10
11
12

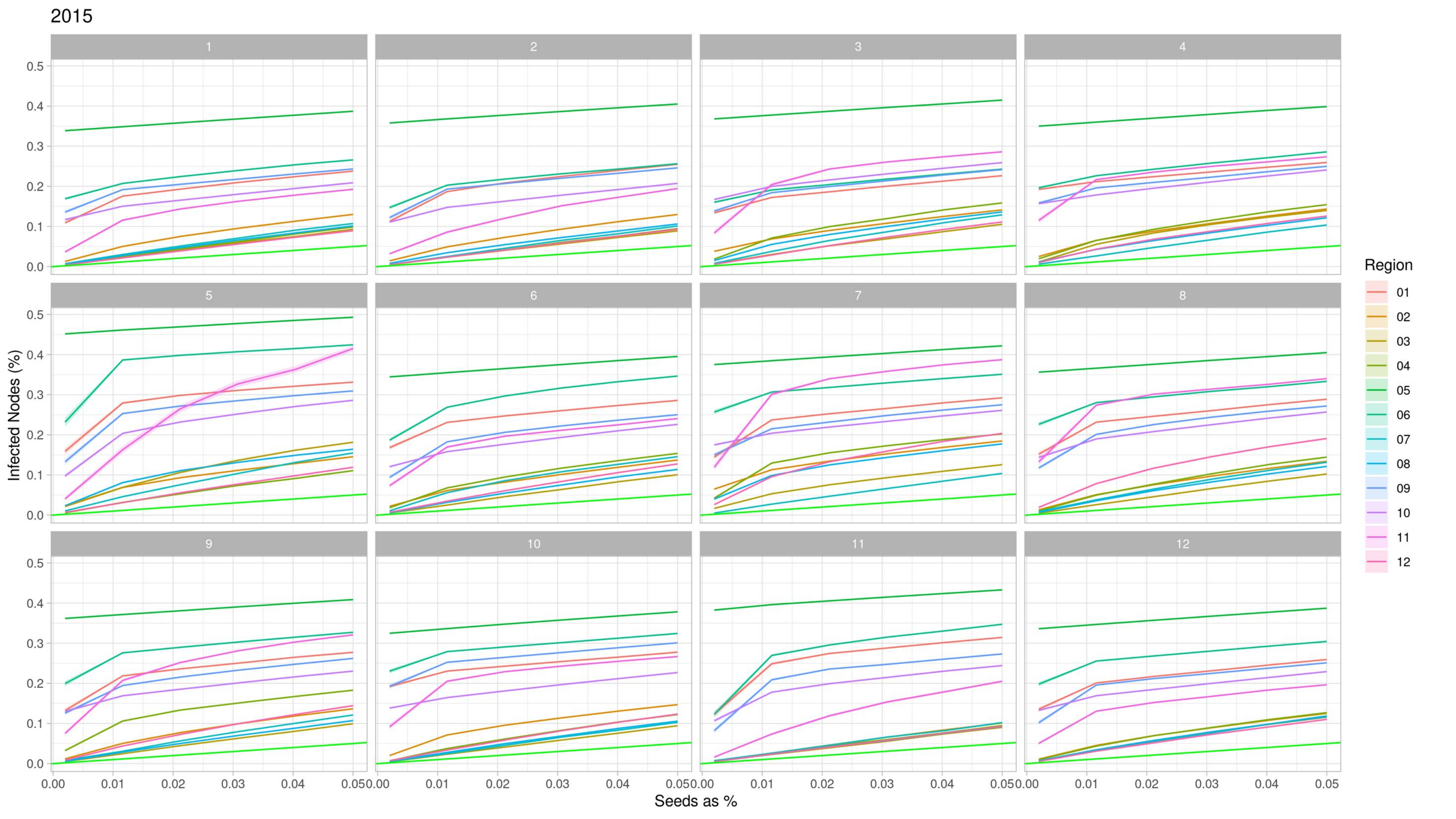
2015
1
2
3
4
5
6
7
8
9
10
11
12
Infected Nodes (%)
Seeds as %
0.0
0.1
0.2
0.3
0.4
0.5
0.00
0.01
0.02
0.03
0.04
0.05
Region
01
02
03
04
05
06
07
08
09
10
11
12

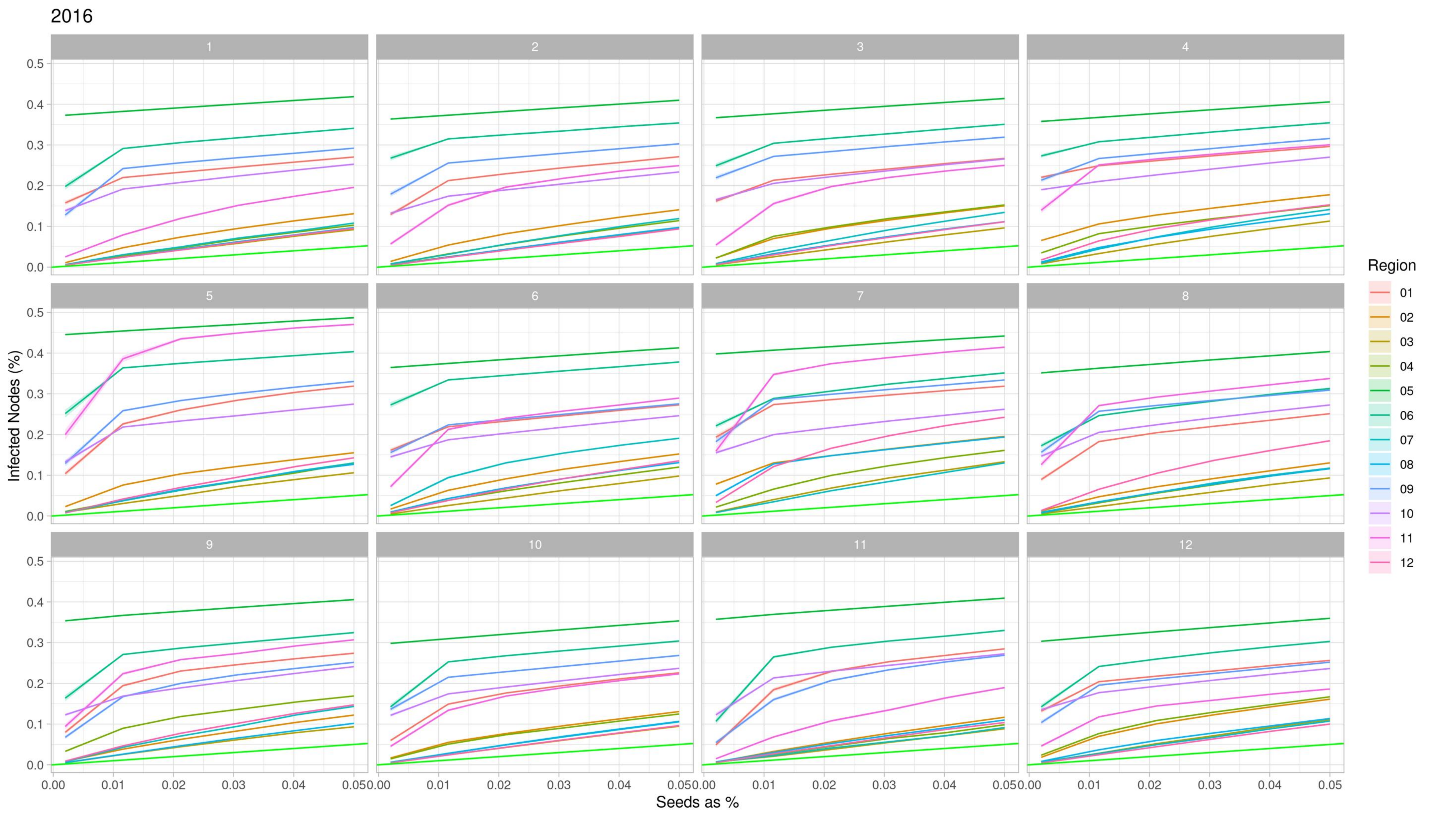
2016
1
2
3
4
5
6
7
8
9
10
11
12
Infected Nodes (%)
Seeds as %
0.0
0.1
0.2
0.3
0.4
0.5
0.00
0.01
0.02
0.03
0.04
0.05
Region
01
02
03
04
05
06
07
08
09
10
11
12

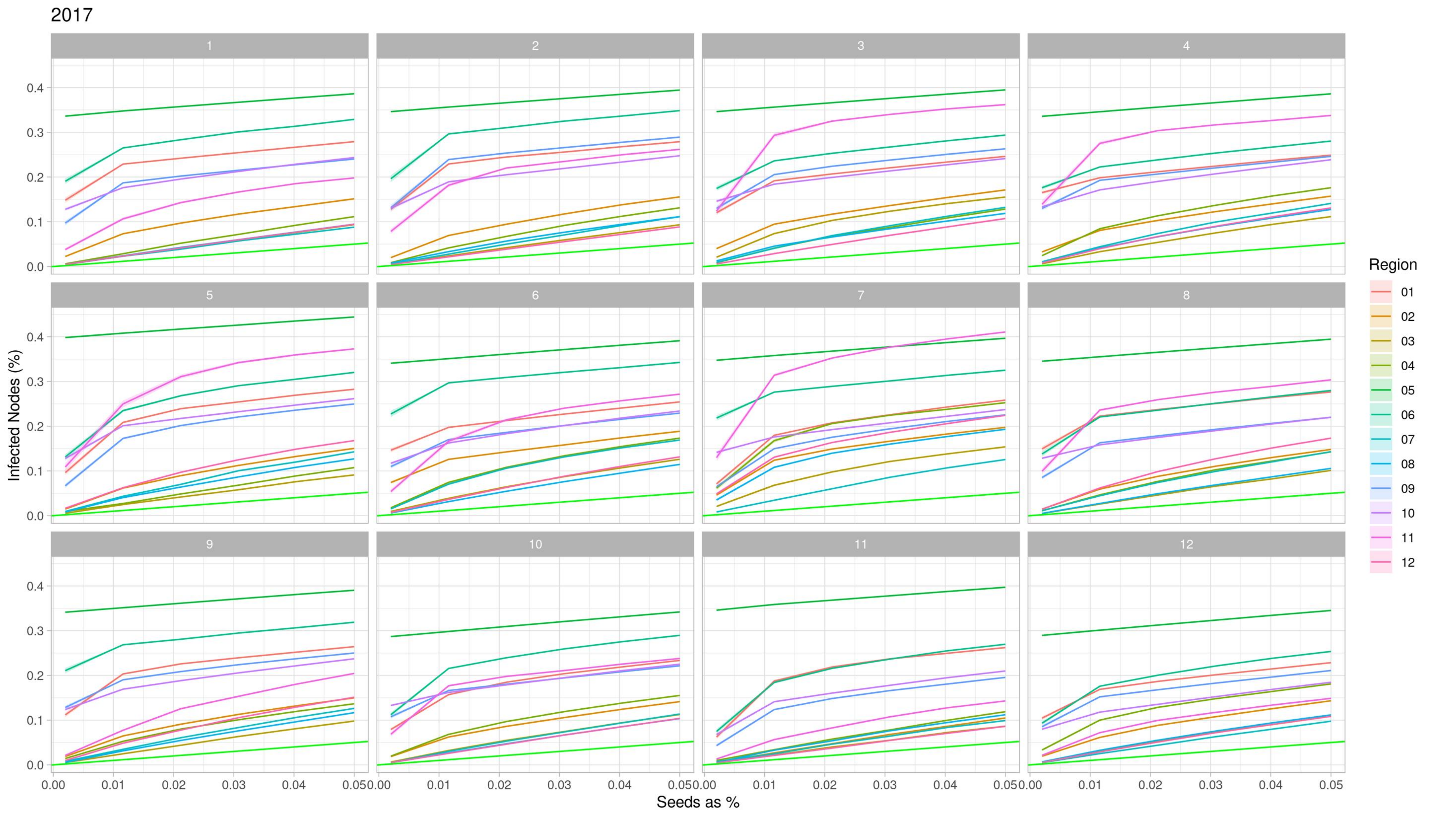

2017
1
2
3
4
5
6
7
8
9
10
11
12
Infected Nodes (%)
Seeds as %
0.0
0.1
0.2
0.3
0.4
0.00
0.01
0.02
0.03
0.04
0.05
Region
01
02
03
04
05
06
07
08
09
10
11
12

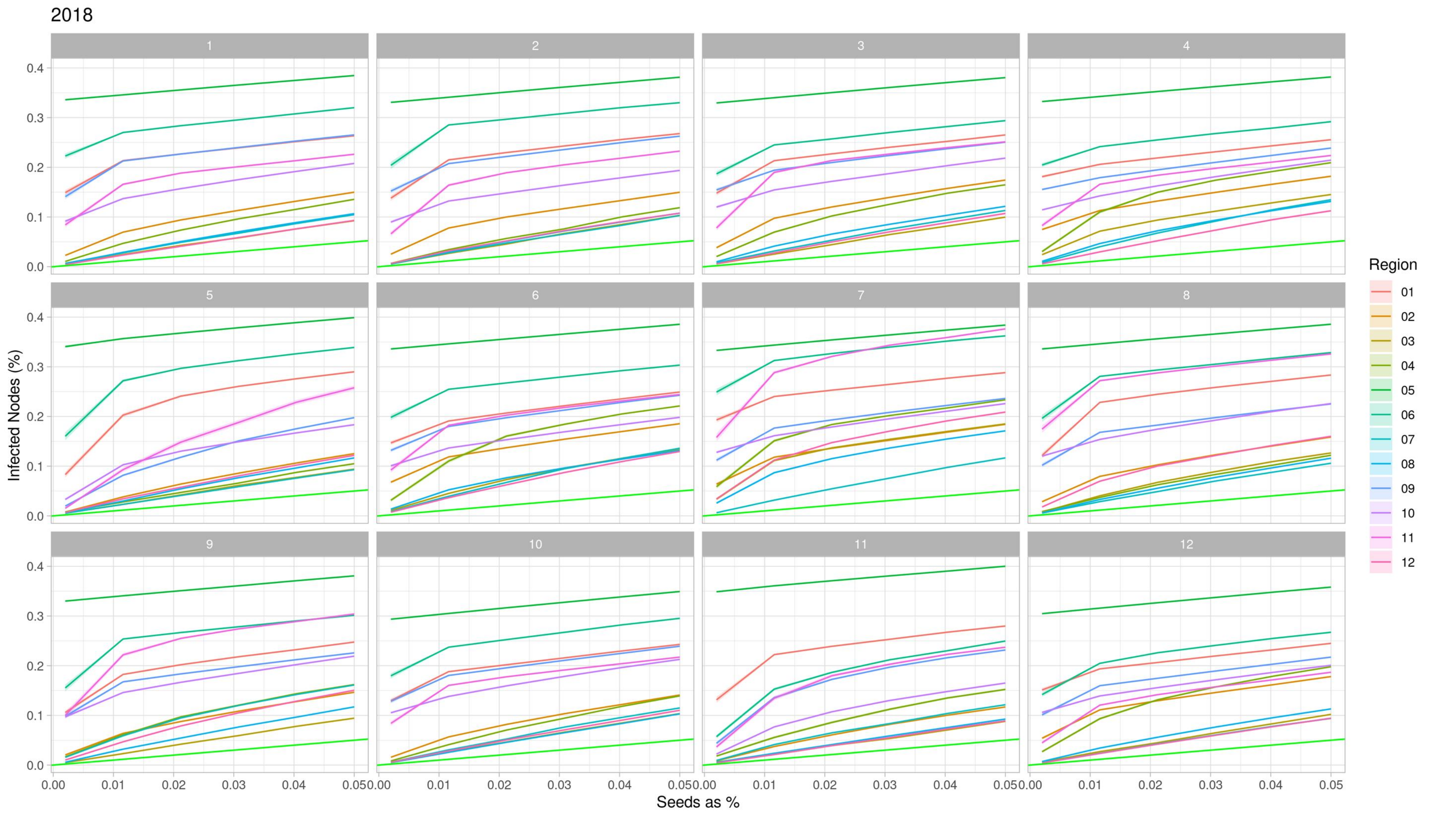
2018
1
2
3
4
5
6
7
8
9
10
11
12
Infected Nodes (%)
Seeds as %
0.0
0.1
0.2
0.3
0.4
0.00
0.01
0.02
0.03
0.04
0.05
Region
01
02
03
04
05
06
07
08
09
10
11
12

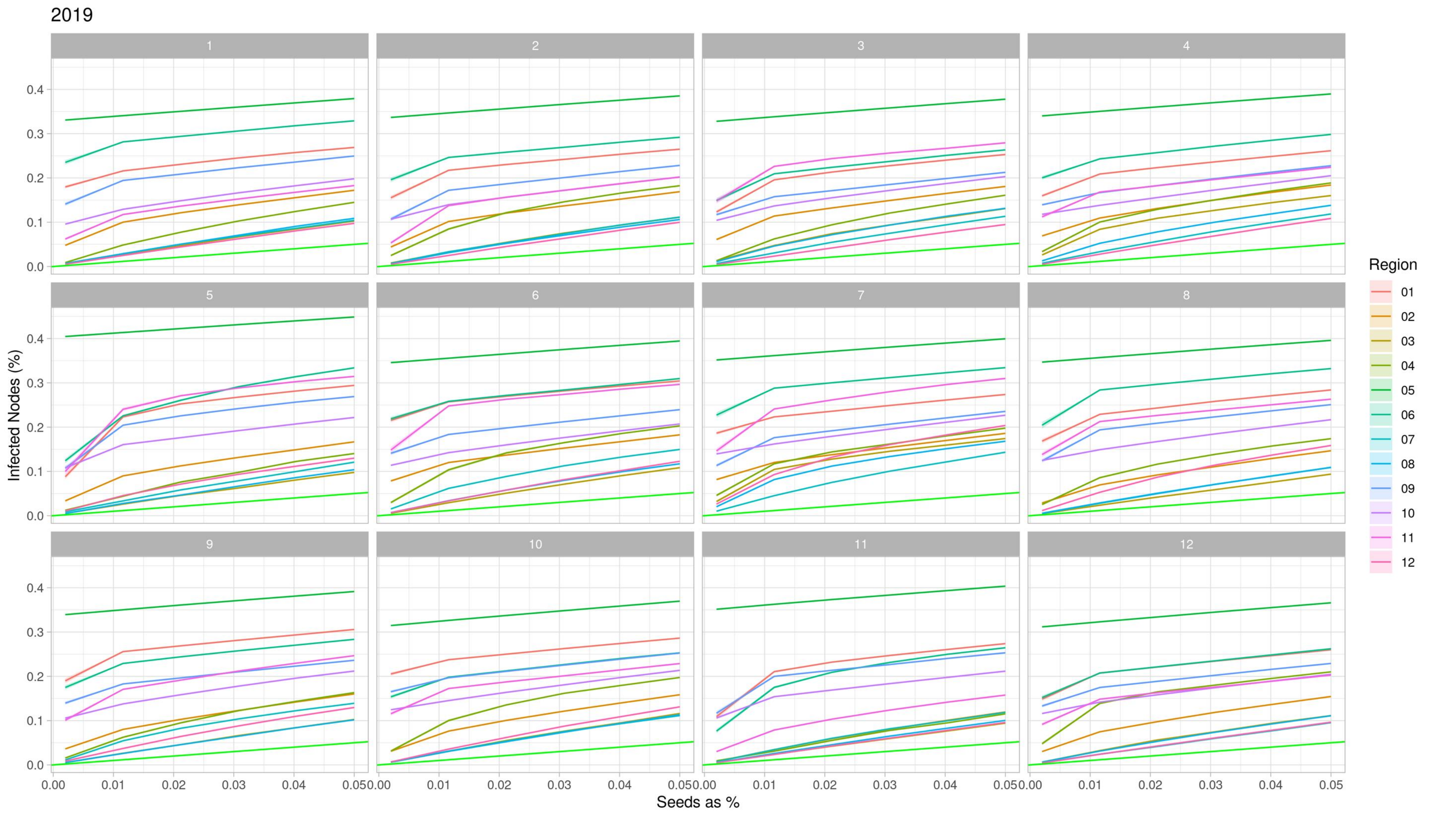
2019
1
2
3
4
5
6
7
8
9
10
11
12
Infected Nodes (%)
Seeds as %
0.0
0.1
0.2
0.3
0.4
0.00
0.01
0.02
0.03
0.04
0.05
Region
01
02
03
04
05
06
07
08
09
10
11
12

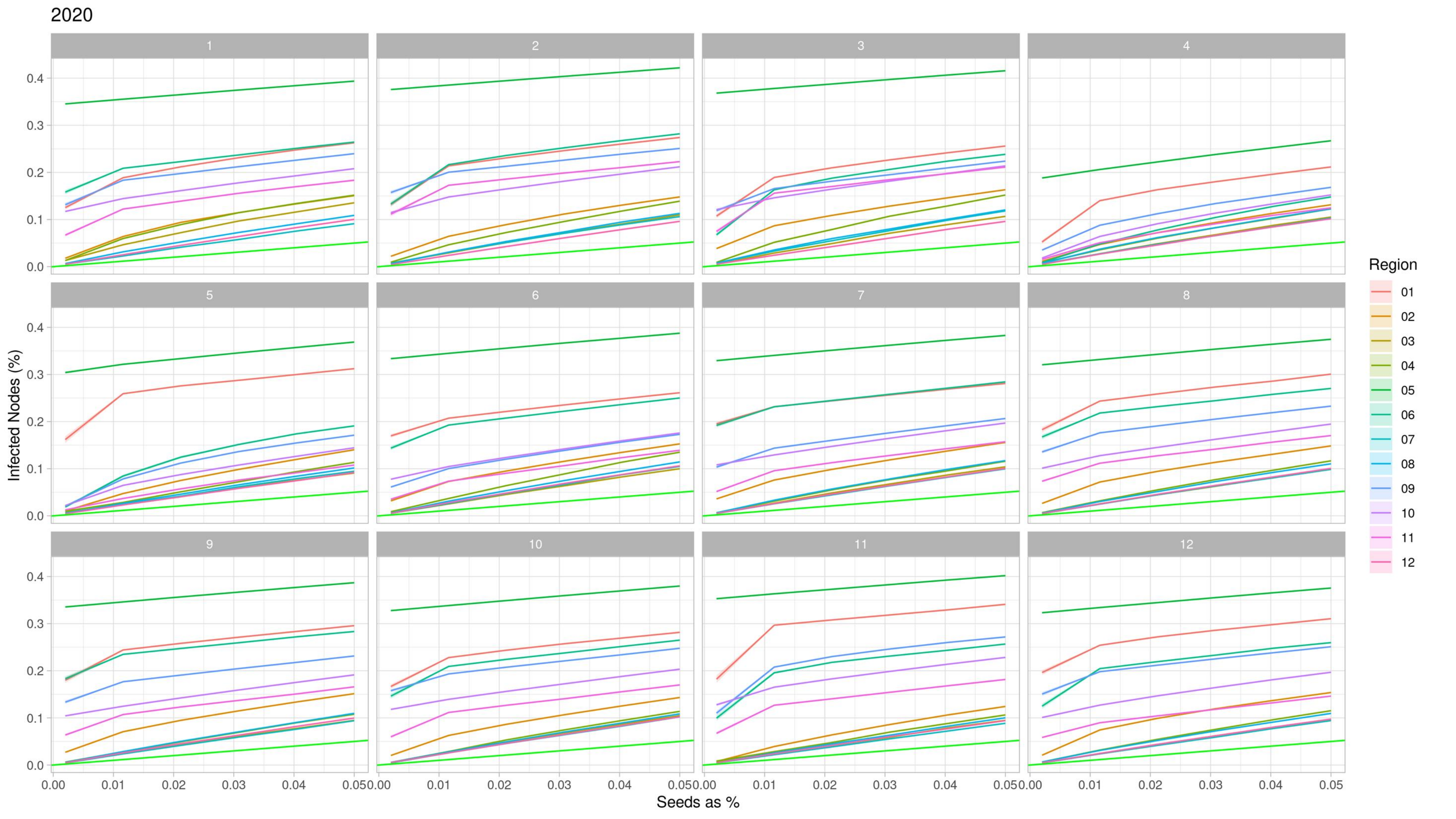

2020
1
2
3
4
5
6
7
8
9
10
11
12
Infected Nodes (%)
Seeds as %
0.0
0.1
0.2
0.3
0.4
0.00
0.01
0.02
0.03
0.04
0.05
Region
01
02
03
04
05
06
07
08
09
10
11
12

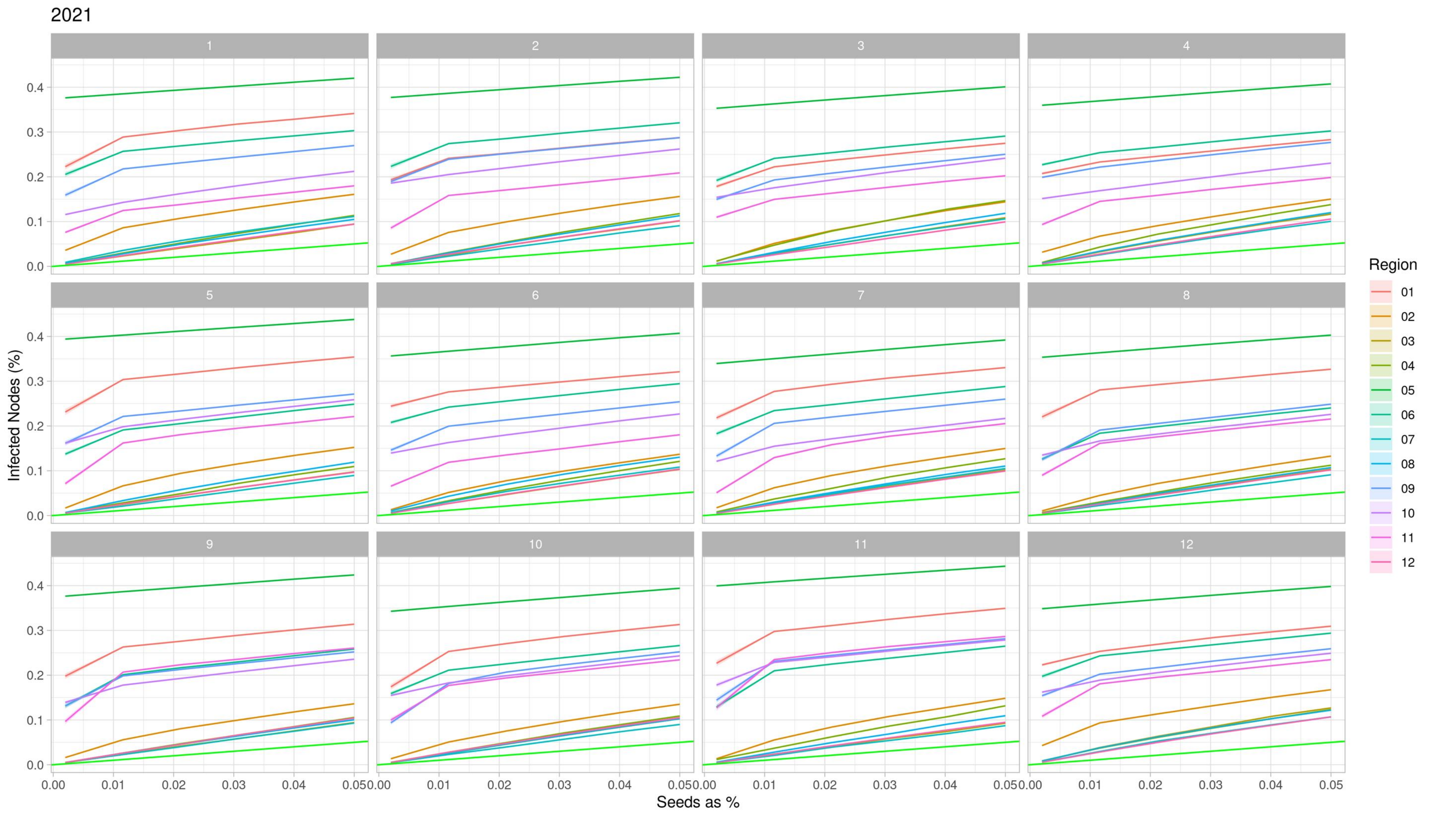
2021
1
2
3
4
5
6
7
8
9
10
11
12
Infected Nodes (%)
Seeds as %
0.0
0.1
0.2
0.3
0.4
0.00
0.01
0.02
0.03
0.04
0.05
Region
01
02
03
04
05
06
07
08
09
10
11
12
