# Supplementary material for: Network vulnerability of cattle movement in Minas Gerais, Brazil, from 2013 to 2022
Source: PLoS One. 2025 Dec 1;20(12):e0317275. doi: 10.1371/journal.pone.0317275 (PMC12668548; doi:10.1371/journal.pone.0317275)
Supplement: S5 Fig — (PDF) [file pone.0317275.s005.pdf]

S5: Correlation Matrix of the network description measures of the network of cattle movement in Minas Gerais, Brazil from 2013 to 2022.

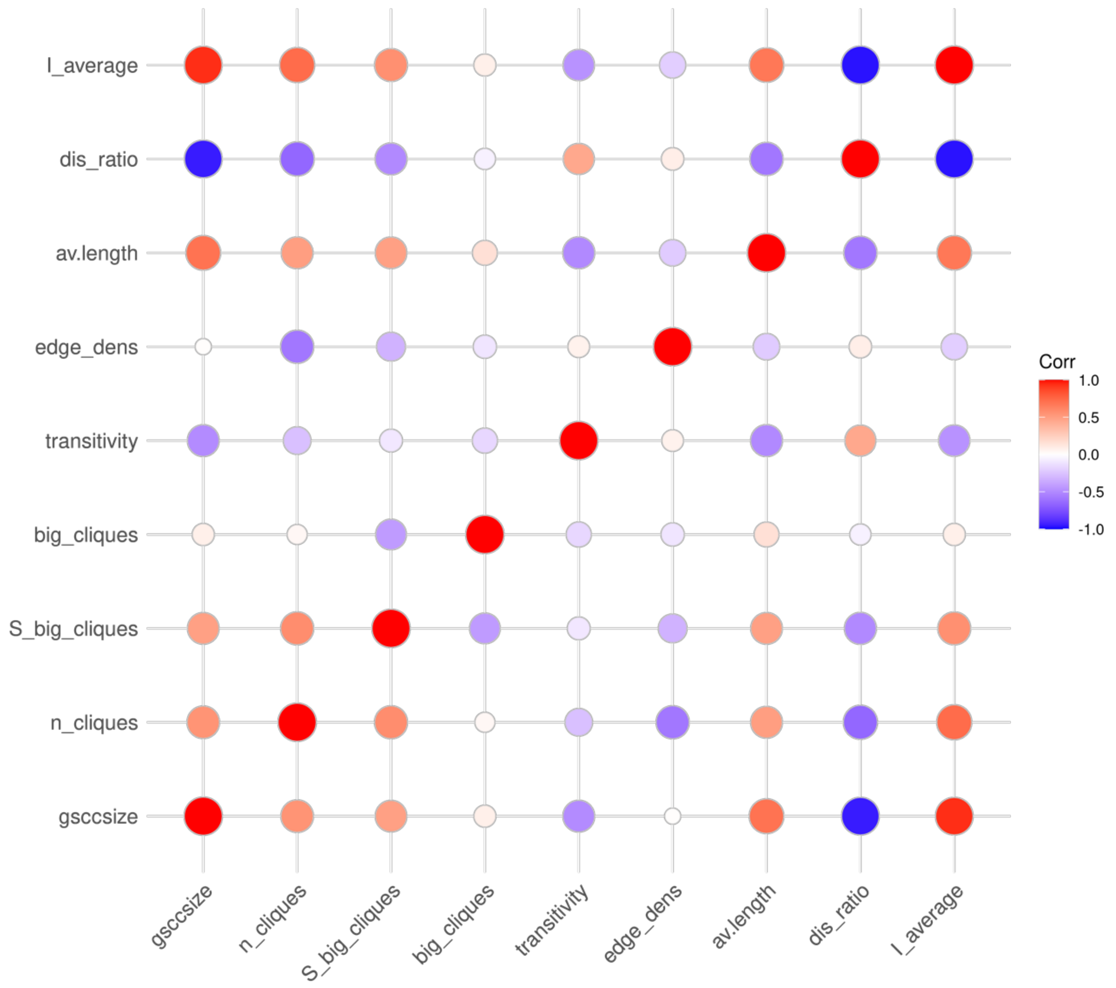

Gsccsize - , N_cliques, S_big_cliques, transitivity, edeges_dens, av.length, dis_ratio, I_average.
