# Supplementary material for: Network vulnerability of cattle movement in Minas Gerais, Brazil, from 2013 to 2022
Source: PLoS One. 2025 Dec 1;20(12):e0317275. doi: 10.1371/journal.pone.0317275 (PMC12668548; doi:10.1371/journal.pone.0317275)

S6: Hubs IN and OUT distribution over hubs ratio and strength ratio. A) Hubs IN distribution in the colored points. Grey scale color of the regions is the hub ratio in each region. B) Hubs OUT distribution in the colored points. Grey scale color of the regions is the hub ratio in each region. C) Hubs IN distribution in the colored points. Grey scale color of the regions is the strength ratio in each region. D) Hubs OUT distribution in the colored points. Grey scale color of the regions is the strength ratio in each region.

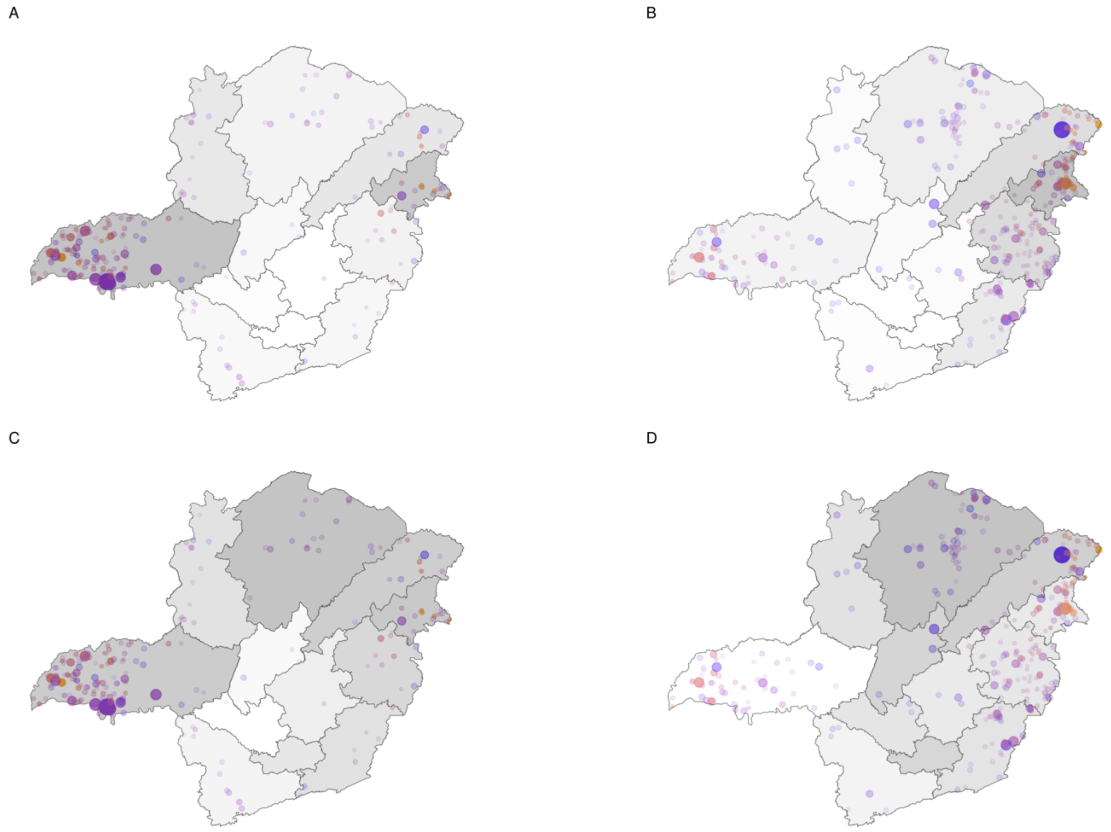

Supplement: S6 Fig — A) Hubs IN distribution in the colored points. Grey scale color of the regions is the hub ratio in each region. B) Hubs OUT distribution in the colored points. Grey scale color of the regions is the hub ratio in each region. C) Hubs IN distribution in the colored points. Grey scale color of the regions is the strength ratio in each region. D) Hubs OUT distribution in the colored points. Grey scale color of the regions is the strength ratio in each region. (PDF) [file pone.0317275.s006.pdf]
