# Supplementary material for: Network vulnerability of cattle movement in Minas Gerais, Brazil, from 2013 to 2022
Source: PLoS One. 2025 Dec 1;20(12):e0317275. doi: 10.1371/journal.pone.0317275 (PMC12668548; doi:10.1371/journal.pone.0317275)
Supplement: Attachment — Submitted filename: Revision_letter_final.docx [file pone.0317275.s008.docx]

**Dr. Lucas D. B. Faria**

Academic Editor,

PLOS ONE

Thank you for your message regarding our manuscript “*Network vulnerability of cattle movement in Minas Gerais, Brazil, from 2013 to 2022*, PONE-D-24-59680*.* We would like to thank you for the appropriate and useful suggestions regarding our manuscript. All the points made by the reviewers are consistent and improved the manuscript.

Please find the enclosed **Responses to Journal Requirements and reviewer #1**.

Sincerely,

Prof. Elaine Maria Seles Dorneles

Departamento de Medicina Veterinária Preventiva,

Universidade Federal de Lavras

Av. Sul UFLA - Aquenta Sol - Lavras, Minas Gerais

Post office box: 3037

Postal Code: 37200-900. Brazil.

elaine.dorneles@ufla.br

Phone: + 55 35 3829 1712

**Journal Requirements:**

**When submitting your revision, we need you to address these additional requirements.**

1. **Please ensure that your manuscript meets PLOS ONE's style requirements, including those for file naming. The PLOS ONE style templates can be found at**

**https://journals.plos.org/plosone/s/file?id=wjVg/PLOSOne_formatting_sample_main_body.pdf and**

**https://journals.plos.org/plosone/s/file?id=ba62/PLOSOne_formatting_sample_title_authors_affiliations.pdf**

Answer: Thank you for the reminder. The files were all updated according to the style requirements.

2. **Please note that PLOS ONE has specific guidelines on code sharing for submissions in which author-generated code underpins the findings in the manuscript. In these cases, we expect all author-generated code to be made available without restrictions upon publication of the work. Please review our guidelines at https://journals.plos.org/plosone/s/materials-and-software-sharing#loc-sharing-code and ensure that your code is shared in a way that follows best practice and facilitates reproducibility and reuse.**

Answer: Thank you for your request. The codes used in this paper are shared here <https://github.com/anninhactrbc/ntw_vulnerability_super_spreaders_susceptible.git>

3. **Thank you for stating the following financial disclosure: The authors would like to thank the Coordenação de Aperfeiçoamento de Pessoal de Nível Superior (CAPES), Fundação de Amparo à Pesquisa do Estado de Minas Gerais (Fapemig) (RED 000132-22) and Conselho Nacional de Desenvolvimento Científico e Tecnológico (CNPq) (402774/2022-1), Brazil, for the financial support. EMSD is thankful to CNPq for her fellowship.**

**Please state what role the funders took in the study. If the funders had no role, please state: ""The funders had no role in study design, data collection and analysis, decision to publish, or preparation of the manuscript.""**

**If this statement is not correct you must amend it as needed.**

**Please include this amended Role of Funder statement in your cover letter; we will change the online submission form on your behalf.**

Answer: We appreciate your request. The correct finacial disclouser were corrected to:

“The authors would like to thank the Coordenação de Aperfeiçoamento de Pessoal de Nível Superior, Brasil (Capes), Fundação de Amparo à Pesquisa do Estado de Minas Gerais (Fapemig) (RED 000132-22) and Conselho Nacional de Desenvolvimento Científico e Tecnológico (CNPq) (402774/2022-1), Brazil, for the financial support. EMSD is thankful to CNPq for her fellowship. The funders had no role in study design, data collection and analysis, decision to publish, or preparation of the manuscript.”

4. **Thank you for stating the following in the Acknowledgments Section of your manuscript: The authors would like to thank the Coordenação de Aperfeiçoamento de Pessoal de Nível Superior, Brasil (Capes), Fundação de Amparo à Pesquisa do Estado de Minas Gerais (Fapemig) (RED 000132-22) and Conselho Nacional de Desenvolvimento Científico e Tecnológico (CNPq) (402774/2022-1), Brazil, for the financial support. EMSD is thankful to CNPq for her fellowship.**

**We note that you have provided funding information that is not currently declared in your Funding Statement. However, funding information should not appear in the Acknowledgments section or other areas of your manuscript. We will only publish funding information present in the Funding Statement section of the online submission form.**

**Please remove any funding-related text from the manuscript and let us know how you would like to update your Funding Statement. Currently, your Funding Statement reads as follows: The authors would like to thankthe Coordenação de Aperfeiçoamento de Pessoal de Nível Superior (CAPES), Fundação de Amparo à Pesquisa do Estado de Minas Gerais (Fapemig) (RED 000132-22) and Conselho Nacional de Desenvolvimento Científico e Tecnológico (CNPq) (402774/2022-1), Brazil, for the financial support. EMSD is thankful to CNPq for her fellowship.**

**Please include your amended statements within your cover letter; we will change the online submission form on your behalf.**

Answer: Thank you for the correction. We removed the financial sources from the acknowledgement section.

5. **In the online submission form, you indicated that all data will be made avaliable uppon request to the corresponding authors.**

**All PLOS journals now require all data underlying the findings described in their manuscript to be freely available to other researchers, either 1. In a public repository, 2. Within the manuscript itself, or 3. Uploaded as supplementary information.**

**This policy applies to all data except where public deposition would breach compliance with the protocol approved by your research ethics board. If your data cannot be made publicly available for ethical or legal reasons (e.g., public availability would compromise patient privacy), please explain your reasons on resubmission and your exemption request will be escalated for approval.**

Answer: Thank you for your request. The data used for this project contain identification details of the premises such as name, latitude and longitude, used in the analysis, the availability of these information would have to be authorized by the owners and that is not possible to acquire in light of the great number of farms in Minas Gerais state, Brazil. Additionally, Brazil has a law Lei Geral de Proteção de Dados Pessoais (LGPD), Lei n° 13.709/2018, that prohibit us to share or even access of some the personal information that was in our data.

6. **We note that Figure 1, 8 and 10 in your submission contain [map/satellite] images which may be copyrighted. All PLOS content is published under the Creative Commons Attribution License (CC BY 4.0), which means that the manuscript, images, and Supporting Information files will be freely available online, and any third party is permitted to access, download, copy, distribute, and use these materials in any way, even commercially, with proper attribution. For these reasons, we cannot publish previously copyrighted maps or satellite images created using proprietary data, such as Google software (Google Maps, Street View, and Earth). For more information, see our copyright guidelines: http://journals.plos.org/plosone/s/licenses-and-copyright.**

**We require you to either (1) present written permission from the copyright holder to publish these figures specifically under the CC BY 4.0 license, or (2) remove the figures from your submission:**

**a. You may seek permission from the original copyright holder of Figure 1, 8 and 10 to publish the content specifically under the CC BY 4.0 license.**

**We recommend that you contact the original copyright holder with the Content Permission Form (http://journals.plos.org/plosone/s/file?id=7c09/content-permission-form.pdf) and the following text:**

**“I request permission for the open-access journal PLOS ONE to publish XXX under the Creative Commons Attribution License (CCAL) CC BY 4.0 (http://creativecommons.org/licenses/by/4.0/). Please be aware that this license allows unrestricted use and distribution, even commercially, by third parties. Please reply and provide explicit written permission to publish XXX under a CC BY license and complete the attached form.”**

**Please upload the completed Content Permission Form or other proof of granted permissions as an ""Other"" file with your submission.**

**In the figure caption of the copyrighted figure, please include the following text: “Reprinted from [ref] under a CC BY license, with permission from [name of publisher], original copyright [original copyright year].”**

**b. If you are unable to obtain permission from the original copyright holder to publish these figures under the CC BY 4.0 license or if the copyright holder’s requirements are incompatible with the CC BY 4.0 license, please either i) remove the figure or ii) supply a replacement figure that complies with the CC BY 4.0 license. Please check copyright information on all replacement figures and update the figure caption with source information. If applicable, please specify in the figure caption text when a figure is similar but not identical to the original image and is therefore for illustrative purposes only.**

**The following resources for replacing copyrighted map figures may be helpful:**

**USGS National Map Viewer (public domain): http://viewer.nationalmap.gov/viewer/**

**The Gateway to Astronaut Photography of Earth (public domain): http://eol.jsc.nasa.gov/sseop/clickmap/**

**Maps at the CIA (public domain): https://www.cia.gov/library/publications/the-world-factbook/index.html and https://www.cia.gov/library/publications/cia-maps-publications/index.html**

**NASA Earth Observatory (public domain): http://earthobservatory.nasa.gov/**

**Landsat: http://landsat.visibleearth.nasa.gov/**

**USGS EROS (Earth Resources Observatory and Science (EROS) Center) (public domain): http://eros.usgs.gov/#**

**Natural Earth (public domain):** [**http://www.naturalearthdata.com/**](http://www.naturalearthdata.com/)

Answer: We appreciate your concern, however the maps produced in this paper were developed by the authors. The based maps used the multipolygon that produce the limit lines of the state and country and it was aquired in a free R package called “geobr”, by Pereira R, Goncalves. _geobr: Download Official Spatial Data Sets of Brazil_. R package version 1.7.0. 2022, cited and referenced in the paper. This package uses free data on geographic information of Brazil and its states that are provided by Instituto Brasileiro de Geografia e Estatistica (IBGE). All information can be found here <https://github.com/ipeaGIT/geobr>.

**Reviewer #1: General Comment**

**This study focuses on analyzing the vulnerability of livestock movement networks in Minas Gerais, Brazil. Such research is particularly valuable for anticipating the spread of animal diseases transmitted directly between animals. The objective is to identify the best strategies to reduce the network's disease vulnerability. According to the results, centrality measures such as out-degree and betweenness seem to be the best strategy. Additionally, the study highlights the most vulnerable regions within the state, which can be helpful for targeted surveillance efforts. However, the study does not fully capture the dynamics of an epidemic.**

**There is a lack of context regarding livestock movements, which would help justify the necessity of this type of study and guide the reader more effectively.**

**Certain sections lack well-organized ideas and sentences, making them difficult to follow. For instance, in the Materials and Methods section, some justifications for specific choices appear later in the text when they would be more helpful earlier.**

**The discussion section should also be improved to provide more context to justify the results (if possible). Additionally, it would be interesting to include perspectives on this study's potential implications and applications.**

**Specific comment**

**Abstract & Introduction**

**Comment 1:**

**Add a sentence at the beginning of the abstract that briefly defines the topic.**

Answer: Thank you for your request. Please see the sentence at the beginning of the abstract.

“*The analysis of networks from cattle movements is an important approach to investigate areas and premises where disease outbreaks can occur and be contained. Vulnerability analysis allows a more profound understanding of the network by combining network measures with the strategic removal of nodes, helping to identify more vulnerable areas and the best metrics to support disease control planning.”*

**Comment 2:**

**The introduction lacks sufficient context about the movements in Brazil, particularly in Minas Gerais. It is strongly recommended that a general overview of these movements and the diseases they transmit be provided, specifically focusing on the study area.**

Answer: We appreciate your suggestion. There is one paper already published describing the network of cattle movement in that region. Even so, we added a phrase in the introduction to give some context, as follow:

“*The network analysis of cattle movement in Minas Gerais was shown elsewhere [14], describing a very connected network with movements more focused in the west and east sides of the state, being the Triângulo Mineiro / Alto do Paranaíba and Vale do Mucuri regions of great emphasis on movements and cattle population [15].”*

**M&M**

**Comment 3:**

**In the Materials & Methods section, adding a subsection titled "Software" and listing all the packages used would be better**

Answer: We appreciate your suggestion. The new section was added to the Material and Methods.

“Software

All analysis were conducted into R software version 4.3.0[19], being the data organized with the packages “readxl” version 1.4.2 [20], “forecast” [21], "stringi" [22] and “tidyverse” [23]. All vulnerability analyses were performed with the packages “tidyverse” [23], “igraph” package [29] and “geobr” package [30]. Additionally, all spatial spreader analyses were performed with the packages “tidyverse” [23], “igraph” package [29], “geobr” package [30] and “geosphere” [32].”

**Comment 4 :**

**It would be better to start the Vulnerability section (M&M) with a brief definition of network vulnerability.**

Answer: We appreciate the suggestion. The definition of the vulnerability and the reason of its use were added to the beginning of the section about this subject.

“*Network vulnerability was defined as the structural potential of a network to propagate an infection starting from a limited number of initially affected nodes, based solely on its topology. This metric estimates how many nodes can be reached through directed links when a given proportion of premises is considered infected.*

*To calculate network vulnerability, we used a custom R function (Vulnerability.table) developed with the igraph and tidyverse packages. The function simulates the reachability of infected nodes by randomly selecting different percentages of seed nodes (ranging from 1% to 30% of all nodes) and measuring the number of unique nodes reachable from them through directed paths. For each seed percentage, the function performs 1,000 simulations and returns the mean and standard deviation of the proportion of nodes reached. Reachability is calculated using ego-networks of radius equal to the network diameter, in “out” mode (i.e., considering all downstream connections). The vulnerability curve is defined by plotting the average proportion of infected nodes (y-axis) against the seed node proportion (x-axis). Higher curves indicate greater vulnerability. In some cases, the area under this curve (AUC) was used as a synthetic vulnerability index. The function also optionally returns a ggplot-based visualization with confidence bands. Then, network vulnerability was defined as the relationship between the average percentage of reachable nodes and the percentage of seed nodes. This approach, based on the reachability from randomly selected nodes using ego-networks, was adapted from methods described by Natale et al. (2011), Keeling and Eames (2005), and Cardenas et al. (2021), where vulnerability is expressed in terms of the system’s structural capacity to propagate infection through network connections*”

**Comment 5:**

**“This procedure was repeated 1000 times for each percentage of seed nodes (0.02%, 0.10%, 0.18%, 0.26%, 0.34%, and 0.50%) to estimate average and standard deviation (SD)”.**

**Do the percentages here correspond to the random percentage of selected nodes?**

**The following paragraph needs to be rewritten or clarified. “To estimate network vulnerability, a defined percentage of nodes was randomly selected as seed nodes and the igraph's ego function was used to calculate the number of nodes reachable downstream from each seed node, expressed as a percentage of the network size. This procedure was repeated 1000 times for each percentage of the seed nodes (0.02%, 0.10%, 0.18%, 0.26%, 0.34%, and 0.50%) to estimate the average and standard deviation (SD)”**

Answer:

Thank you for your observation. Yes, the listed percentages (0.02%, 0.10%, etc.) refer to the proportion of nodes that are randomly selected as seed nodes in each simulation. To address your concern, we have clarified the paragraph in the Methods section accordingly. We believe that, with the improved definition of network vulnerability introduced at the beginning of the section (as per your previous comment), the revised paragraph is now clearer.

The text in the manuscript has been updated as follows:

“To estimate network vulnerability, a fixed proportion of nodes was randomly selected as seed nodes (i.e., premises assumed to be infected). For each proportion tested (0.02%, 0.10%, 0.18%, 0.26%, 0.34%, and 0.50% of all nodes), 1,000 independent simulations were performed using the defined vulnerability metric, to estimate the average vulnerability, along with its standard deviation (SD). ”.

**Comment 6:**

**Reference for this definition?**

**“Network vulnerability was then defined as the relationship between the average percentage of reachable nodes and the percentage of seed nodes. »**

Answer: Thank you for raising this point. As suggested in your previous comments, we have revised the section on vulnerability analysis to include a more formal definition and clearer methodological explanation. The revised paragraph now clarifies that network vulnerability is computed as the proportion of nodes reached from randomly selected seed nodes, and we refer to previous works that inspired this topological diffusion-based approach. Specifically, our method aligns conceptually with approaches used in:

- Natale et al. (2011) – where disease flow centrality and reachability from seed nodes are used to assess vulnerability in cattle trade networks.
- Keeling & Eames (2005) – which discusses infection spread in networks and the influence of structure on propagation potential.
- Cardenas et al. (2021) – which uses ego-network reachability and network metrics to quantify spatio-temporal vulnerability in animal movement networks.

**Comment 7:**

**This section should be moved to the part where you discuss the data. In this paragraph, you only need to specify that this was applied to the aggregated data. Additionally, in the data section, it would be helpful to include more details about what was used to reconstruct the networks.**

**“The datasets were grouped by local of origin, local of destination, region (origin and**

**destination) and month per year to create a list of the network objects, from which the**

**vulnerability R function (S1 function) was applied.”**

Answer: Thank you for your suggestion. We removed the pointed-out sentence and used in the “Data source and description” section. ´

**Comment 8:**

**Repetition**

**“The vulnerability of each region was plotted for each month of the year, from 2013 to 2022”**

**“was plotted per month and region of each year”**

Answer: We appreciate your comment. We changed the word “plotted” in the first part for “mapped” to avoid repetition.

**Comment 9**

**Maybe you should mention it first, in the part where you talk about vulnerability.**

**“This vulnerability indicator provides an estimation of how many nodes could be affected by an outbreak once one or more nodes in the region become exposed or infected.”**

Answer: Thank you for your suggestion. We used the definition of the vulnerability function and its applicability in the beginning of the section in a similar statement, therefore, we respectfully would like to keep the sentence in the Spatial Spreader section to contextualize the implementation of the function.

**Comment 10:**

**Suggestion: In the Vulnerability section, you can add two sub-sections: Local Vulnerability and Global Vulnerability.**

Answer: Thank you for your suggestion. We renamed the sections as suggested.

**Comment 11**

**Why was 2022 chosen for the distance evaluation?**

**The answer is provided later; I just saw it. It might be better to justify this point when you first mention it.**

Answer: Thank you for your comment. The justification was moved to this section to better clarify this point.

**Comment 12**

**Justify why in-degree, out-degree and betweenness were selected as centrality measures in the tested scenarios (include references or other justifications). Why not eigenvector, for exemple?**

Answer: Thank you for your comment. The selection of in-degree, out-degree, and betweenness centrality was based on their widespread use and demonstrated effectiveness in risk-based surveillance and intervention strategies in livestock movement networks. These metrics capture different but complementary aspects of node importance. In-degree reflects the susceptibility of a node, as a higher number of incoming movements implies increased exposure to potential infection sources. Out-degree is associated with the spreading potential of a node, indicating how many other nodes it can directly affect. Betweenness centrality identifies nodes that act as bridges in the network, which are key for targeting interventions to fragment the system and slow down disease transmission. These measures have been widely used in veterinary epidemiology and shown to be effective in identifying critical nodes for surveillance, as reported in: Natale et al., 2009; Nöremark et al., 2011; Cardenas et al., 2021; Hasahya et al., 2023. About eigenvector centrality, we decided not to include it in our analysis because this measure tends to overemphasize nodes that are connected to other central nodes, regardless of the actual flow or direction of movements. In livestock movement networks, which are directed and weighted, eigenvector centrality may be less informative or even misleading, as it does not distinguish between incoming and outgoing links and may correlate poorly with epidemiological relevance in such systems (see Lentz et al., 2016). Therefore, we prioritized centrality metrics with clearer interpretability in the context of directional disease spread. We added the following sentence in the section:

“*In-degree, out-degree and betweenness centrality were selected for the intervention scenarios because they are widely used in veterinary epidemiology and have shown effectiveness in identifying high-risk nodes in livestock movement networks. These measures are especially relevant in directed movement networks, where flow direction matters [2,4,8,10].”*

**Suggestions: It would be interesting to test multiple percentages to determine how many nodes need to be removed to achieve an optimal result. I suppose the higher the percentage, the more optimal the outcome. But what about the range between 1% and 5%? In other words, is removing 1% of nodes sufficient, or do we need to go up to 5%?**

Answer: Thank you for this suggestion. We agree that testing a range of percentages to identify the optimal number of nodes for removal would be an interesting extension, especially from a cost-effectiveness perspective. However, the main goal of our analysis was to compare which centrality measure is most effective in reducing network vulnerability, not to define the optimal percentage of removed nodes. Our intent, as shown in Fig. 9, was to evaluate relative effectiveness across measures under a fixed and practical scenario, rather than to optimize the intervention size. Further analysis using a broader range of removal percentages (e.g., 1–10%) could indeed be explored in future work focused on operational decision-making and cost-benefit evaluation.

**Comment 13:**

**You need to read this subsection in its entirety to understand why the year 2022 was selected. Find a way to rewrite it so that the reader can grasp why the transition is made from the years 2013-2022 to 2022. Justify this at the beginning.**

Answer: We appreciate your comment. Please see the “in 2022” was added at the end of the first phrase of the topic.

**Results**

**Comment 14:**

**A figure is needed to illustrate this.**

**“The vulnerability analysis per region and per month of the network from cattle movement in Minas Gerais, Brazil, from 2013 to 2022, showed the Triângulo Mineiro / Alto Paranaíba (05) as the most vulnerable except in July of 2017 and 2022, when Campo das Vertentes (11) region showed higher vulnerability, based on the threshold greater than 0.02% of seed used for the analysis. Over the years, 2020 stood out as the year with the lowest vulnerability across all regions in every month, compared with the years before and after. The regions that appeared less vulnerable throughout the months in all years were Jequitinhonha (03) and Metropolitan of Belo Horizonte (07)”**

Answer: Thank you for your solicitation. The figure in question is shown in the supplementary material and could not be present in the body of the paper because that would mean 11 composed figures which each one would have 12 squares for each month of each year with 12 lines in each square for each region of the state. Therefore, to describe some important changes over the years we wrote it in the text, although the reader needs to access the supplementary material to see the yearly variations in detail.

**Comment 15:**

**It is better to put this sentence in method “The year 2022 was chosen for the super spatial hubs analysis, since the previously years showed the same pattern in vulnerability and similar behavior in the network analysis performed elsewhere [19] and was the more recent year of the dataset”**

Answer: Thank you for your suggestion. Indeed, moving this explanation to the M&M made the text better.

**Comment 16:**

**What does Betweenness 21 correspond to in Figure 9?**

Answer: Thank you for your question. Betweenness 21 correspond to the removal of the 5% of the nodes from 2021 that presented greater betweenness in the data of 2022 to see if the indeed betweenness was the best measure to be used even when considering the values and nodes from the year before. To clarify in the text, we added that the node removal occurs in the 2022 network at the end of the risk-based section, and we better explained in the Fig.9 description. Therefore, the Fig 9 name and description is as follow:

“F**ig 9: Risk-Based Assessment of the Triângulo Mineiro / Alto Paranaíba Region.** Reduction in vulnerability when 5% of nodes are eliminated based on higher betweenness centrality, higher in-degree, higher out-degree, and randomly for the year 2022 (A). Impact on vulnerability when nodes are removed from 2022 considering the nodes with greater betweenness according to the network structure of the previous year (2021) (Betweenness 21) and the other measures are as shown in graph A (B).”

**Discussion:**

**Rewrite this sentence: “The adopted vulnerability measure considers the number of nodes that can be reached from randomly chosen nodes within the network. Assuming that the chosen nodes are disease seeds, this measure provides an estimate of the epidemic due to the network's structure”**

Answer: Thank you for your request, it improved the quality of the text. Please see below how the is the new phrase:

“*The adopted vulnerability measure provides an estimate of an epidemic spread due to the network's structure [33], by considering the number of nodes that can be reached from randomly chosen seed nodes within the network.*”

**Comment 17:**

**I thought that out-degree and betweenness were the most important measures.**

**“The analysis demonstrated that by applying centrality measures, such as in-degree, out-degree, and betweenness, to identify and exclude certain nodes, the region's vulnerability could be significantly reduced”**

Answer: Thank you for your comment. Indeed, the betweenness was the best measure followed by out-degree and in-degree. The phrase was changed to clarify this point. The phrase is now as follow:

“*The analysis demonstrated that by applying centrality measures, such as betweenness, out-degree and in-degree, in that order, to identify and exclude certain nodes, the region's vulnerability could be significantly reduced (Fig 9).”*
