# Supplementary material for: Network vulnerability of cattle movement in Minas Gerais, Brazil, from 2013 to 2022
Source: PLoS One. 2025 Dec 1;20(12):e0317275. doi: 10.1371/journal.pone.0317275 (PMC12668548; doi:10.1371/journal.pone.0317275)
Supplement: Attachment — Submitted filename: second_review_latter.docx [file pone.0317275.s009.docx]

**Dr. Lucas D. B. Faria**

Academic Editor,

PLOS ONE

Thank you for your message regarding our manuscript “*Network vulnerability of cattle movement in Minas Gerais, Brazil, from 2013 to 2022*, PONE-D-24-59680*.* We would like to thank you for the appropriate and useful suggestions regarding our manuscript. All the points made by the reviewers are consistent and improved the manuscript.

Please find the enclosed **Responses to reviewer #2**.

Sincerely,

Prof. Elaine Maria Seles Dorneles

Departamento de Medicina Veterinária Preventiva,

Universidade Federal de Lavras

Av. Sul UFLA - Aquenta Sol - Lavras, Minas Gerais

Post office box: 3037

Postal Code: 37200-900. Brazil.

elaine.dorneles@ufla.br

Phone: + 55 35 3829 1712

**Figure 9 Legend: The explanation for "Betweenness 21" is now in the main text, which is good. Ensure the final legend for Figure 9B is exceptionally clear, perhaps phrasing it as:**

**"Impact on vulnerability when nodes are removed from the 2022 network based on the betweenness centrality values calculated from the 2021 network (Betweenness 21), compared to other strategies."**

Answer: We appreciatte your comments and your suggestion. The change was added to the legend of Figure 9B as requested.

**Data Availability Statement: The justification for restricted data access due to LGPD regulations is completely valid and common for this type of data. The statement is clear. It might be marginally improved by explicitly stating that the data can be accessed for research purposes upon reasonable request and approval from the Instituto Mineiro de Agropecuária (IMA), which is implied but could be slightly more direct. E.g., "Data are available from the Instituto Mineiro de Agropecuária (IMA) (contact: ima@ima.mg.gov.br) for researchers who meet the criteria for access to confidential data, as required by Brazilian law (Lei Geral de Proteção de Dados Pessoais - LGPD, Lei n° 13.709/2018)."**

Answer: Thank you for the improvement made to our Data Availability Statement. We will include as suggested in the website.
